# Joint and fascial chronic graft-vs-host disease: correlations with clinical and laboratory parameters

**DOI:** 10.3325/cmj.2016.57.266

**Published:** 2016-06

**Authors:** Tamara Vukić, Sean Robinson Smith, Dina Ljubas Kelečić, Lana Desnica, Ema Prenc, Dražen Pulanić, Radovan Vrhovac, Damir Nemet, Steven Z. Pavletic

**Affiliations:** 1 Department of Rehabilitation and Orthopaedic Aids, University Hospital Center Zagreb, Zagreb, Croatia; 2 Department of Physical Medicine and Rehabilitation, University of Michigan, Ann Arbor, MI, USA; 3 Clinical Unit for Clinical Nutrition, Department of Internal Medicine, University Hospital Center Zagreb, Zagreb, Croatia; 4 Division of Hematology, Department of Internal Medicine, University Hospital Center Zagreb, Zagreb, Croatia; 5 Croatian Cooperative Group for Hematologic Diseases (CroHem), Zagreb, Croatia; 6 University of Zagreb School of Medicine, Zagreb, Croatia; 7 Faculty of Medicine Osijek, J.J. Strossmayer University of Osijek, Osijek, Croatia; 8 Experimental Transplantation and Immunology Branch, National Cancer Institute, Center for Cancer Research, National Institutes of Health, Bethesda, MD, USA

## Abstract

**Aim:**

To determine if there are correlations between joint and fascial chronic graft-vs-host disease (cGVHD) with clinical findings, laboratory parameters, and measures of functional capacity.

**Methods:**

29 patients were diagnosed with cGVHD based on National Institutes of Health (NIH) Consensus Criteria at the University Hospital Centre Zagreb from October 2013 to October 2015. Physical examination, including functional measures such as 2-minute walk test and hand grip strength, as well as laboratory tests were performed. The relationship between these evaluations and the severity of joint and fascial cGVHD was tested by logistical regression analysis.

**Results:**

12 of 29 patients (41.3%) had joint and fascial cGVHD diagnosed according to NIH Consensus Criteria. There was a significant positive correlation of joint and fascial cGVHD and skin cGVHD (*P* < 0.001), serum C3 complement level (*P* = 0.045), and leukocytes (*P* = 0.032). There was a significant negative correlation between 2-minute walk test (*P* = 0.016), percentage of cytotoxic T cells CD3+/CD8+ (*P* = 0.022), serum albumin (*P* = 0.047), and Karnofsky score (*P* < 0.001). Binary logistic regression model found that a significant predictor for joint and fascial cGVHD was cGVHD skin involvement (odds ratio, 7.79; 95 confidence interval 1.87-32.56; *P* = 0.005).

**Conclusion:**

Joint and fascial cGVHD manifestations correlated with multiple laboratory measurements, clinical features, and cGVHD skin involvement, which was a significant predictor for joint and fascial cGVHD.

Chronic graft-vs-host disease (cGVHD) is a major late complication of allogeneic hematopoietic stem cell transplantation (alloHSCT), typically occurring within three years post-transplant and affecting approximately 30%-50% of allogeneic transplant survivors (1-5). Its effects can be devastating, including high morbidity and disability, and it is the leading cause of non-relapse mortality (5). cGVHD is distinct from acute GVHD, which manifests earlier in the post-transplant course, with characteristic skin and gastrointestinal symptoms, and affects multiple organ systems including the joints and fascia, skin, eyes, mouth, lung, liver, gastrointestinal tract, and genital tract in female patients (5). cGVHD occurs because the recipient’s immune system recognizes the donor’s tissues as foreign, causing inflammation and fibrosis in highly mitotic areas (6). Potential risk factors for cGVHD development include major human leukocyte antigen (HLA) allele mismatch between donor and recipient, T-cell replete graft, the use of peripheral blood as the stem cell source, donor lymphocyte infusion, older recipient age, the use of a female donor for a male recipient, and prior acute GVHD (7). Although cGVHD can involve many organ systems, joint and fascial involvement is relatively common and can cause significant functional impairment (8). Caused by inflammation of the fascia, including an eosinophilic component, it may manifest as joint stiffness, erythema, edema, restricted range of motion (ROM), arthralgia, and rarely arthritis or synovitis (5,6,8). Joint and fascial manifestations can be clinically detectable when inflammation and fibrosis arise in deep tissues (deep sclerosis/fasciitis) or skin overlying joints (superficial sclerosis) (5,6,8). Widespread sclerosis may result in joint contractures and severe limitation of function, and common sites of involvement include the hands/wrists, shoulders, elbows, and ankles (6,8).

Despite the potential for functional impairment in persons with joint and fascial cGVHD, little is known about the correlation of joint and fascial cGVHD with other clinical and laboratory manifestations of cGVHD, and about the overall physical performance in persons with joint and fascial cGVHD (9). Some studies have previously looked at overall physical function in persons with cGVHD, which includes joint involvement (6,10), or attempted to predict joint and fascial cGVHD onset with laboratory data (11), but no study to date has determined the correlations between laboratory data, functional impairment, and joint and fascial cGVHD. In this study, we investigated if there was a correlation between laboratory data, including markers of inflammation and of immune system reconstitution after alloHSCT, clinical and physical manifestations of cGVHD, including those that affect other organ systems, and overall physical function in persons with joint and fascial cGVHD. Knowledge about these correlations may lead to a better understanding of the pathophysiology, earlier detection of the disease, and improved diagnosis and management.

## Patients and methods

Data for this descriptive follow-up study were gathered from 32 patients who were suspected of having developed cGVHD after alloHSCT. This study is a part of a larger protocol entitled “Clinical And Biological Factors Determining Severity and Activity of Chronic Graft-versus-Host Disease after Allogeneic Hematopoietic Stem Cell Transplantation“ which studied the natural history of cGVHD and evaluated patients according to the NIH Consensus Criteria (5). The protocol involved the collection of comprehensive demographic, historical, laboratory, clinical, histopathologic, and imaging data. The research was approved by the Ethics Committee of University Hospital Centre Zagreb and University of Zagreb School of Medicine and all participants or their guardians gave written informed consent in accordance with the Declaration of Helsinki.

Patients were enrolled in this study from October 2013 to October 2015 and evaluated at the University Hospital Centre Zagreb. For each patient NIH cGVHD (0-3) organ-specific scores for 8 organs (skin, eye, mouth, liver, gastrointestinal tract, lungs, joint/fascia, and female genital tract) were assigned by specialists for a specific area after examining every patient. NIH global score was graded as “mild” (1-2 organs with scores 1), “moderate” (more than 2 organs with score 1, any score 2, or lung score 1), or “severe” (any score of 3 or lung score 2) (5). Patients’ performance status was measured with Karnofsky score, a decile scale that classifies patients based on functional impairment (12).

Joint involvement was determined in accordance with written testing procedures and included a measurement of upper and lower body range of motion (ROM), dominant and non-dominant hand grip strength (HGS), and walking assessment (13-18). All measures were obtained in a single patient visit. To assess the severity of joint and fascia involvement, the NIH 0-3 point joint and fascia scale that scores a composite of tightness, ROM and activities of daily living (ADL) was used (Table 1) (5). Joint and fascial cGVHD was diagnosed if the patient had NIH joint and fascia score ≥1 (5). Active assisted ROM measurements were performed in the supine position using a standard goniometer, and all measures were compared with predicted values for each individual joint established by the American Academy of Orthopaedic Surgeons (13). Patients were examined thoroughly by a rehabilitation medicine specialist and detailed medical history was taken to exclude any history of a previous trauma, infection, neuromuscular disorder, or any type of connective tissue disease that could affect patients’ ROM independent of cGVHD. No patients had these conditions, which would have excluded them from the study group. To determine the severity of joint and fascia involvement, the joint with the greatest restriction in ROM was used in the analysis for each patient. The Photographic Range of Motion (P-ROM) scale was also used in this study (19). P-ROM scale captures ROM separately for shoulders, elbows, wrists/fingers, and ankles in a series of images. P-ROM total score is the sum of scores in all 4 joints with a maximum of 25 points. Lower scores indicate more limited ROM. P-ROM scale only considers ROM limitations and does not include tightness or ADLs, which is different from the NIH joint/fascia scale (5,19). The NIH joint/fascia scale and P-ROM scale are validated scales for assessing joint and fascial manifestations (5,6,19). NIH joint/fascia scale was developed to evaluate joint and fascial cGVHD manifestations for baseline and cross-sectional studies, but longitudinal changes may also be evaluated with this scale (5,6). P-ROM scale has been validated to be used in longitudinal studies and was more sensitive to perceived joint worsening among cGVHD joint/fascia manifestations than the NIH joint/fascia scale (6).

**Table 1:** NIH joint/fascia scale for cGVHD*

NIH joint/fascia scale (range, 0-3)
0: No symptoms
1: Mild tightness of arms or legs, normal or mild decreased ROM AND not affecting ADL
2: Tightness of arms or legs OR joint contractures, erythema thought due to fasciitis, moderate decrease ROM AND mild to moderate limitation of ADL
3: Contracture WITH significant decrease of ROM AND significant limitation of ADL (unable to tie shoes, button shirts, dress self-etc.)

*ADL – activities of daily living; cGVHD – chronic graft-vs-host disease; NIH – National Institutes of Health; ROM – range of motion.

Patient walking assessment was performed by a 2-minute walk test, which was used instead of the 6-minute walk test (20,21). This was done because 2-minute walk test is a part of the NIH Consensus Criteria (15), and numerous studies support the construct validity and responsiveness of the 2-minute walk test as a measure of functional capacity in other chronically ill populations (15,20,21). In 2-minute walk test the patient is instructed to walk a 15-m course (7.5 m each direction) with 180 degree turns at each end, and the total distance covered in two minutes is recorded (16). HGS for hand strength evaluation was measured for dominant and non-dominant hand (18), and was performed three times for each hand with small intervals between using a hand hydraulic dynamometer (18) (Jamar Hydraulic Hand Dynamometer; Sammons Preston Rolyan, Chicago, IL, USA).

Laboratory studies were performed at the time of clinical evaluation and blood samples were submitted to routine laboratory analysis at the Department of Laboratory Medicine at the University Hospital Centre Zagreb. Laboratory tests were ordered to identify which laboratory inflammation markers are associated with joint and fascial cGVHD involvement. Previous studies identified a number of laboratory indicators of inflammation (C-reactive protein, white blood count, absolute neutrophil count, platelets, and albumin) that may be affected in patients with moderate or severe cGVHD compared with non-cGVHD transplanted patients (9). Some studies have proposed eosinophilia, CD3 T-cell dose in the graft, and positive antinuclear antibodies (ANAs) as markers of sclerotic-type chronic GVHD (ScGVHD) (22,23), while other have proposed elevated C3 complement and higher platelet count (11). This suggests on-going tissue inflammation in this patient population, potentially evaluated with these laboratory measurements (9,11,22,23).

### Statistical analysis

Categorical data are presented with absolute (N) and relative (%) frequencies and compared using Fisher exact test or Fisher-Freeman-Halton exact test of independence when the contingency table is larger than 2 × 2, and numerical data are presented with median and interquartile range values and compared using Mann-Whitney U test. The normality of distribution was tested using Kolmogorov-Smirnov test. Factors potentially associated with joint and fascial cGVHD were first screened as follows: Spearman rank correlation was used to determine the correlation between continuous variables and Kendall tau to determine the correlation between nominal variables and joint and fascial cGVHD. Correlations were interpreted as follows: rho >0.6 as a strong correlation; 0.3<rho <0.6, as a moderately strong correlation, and rho <0.3 as a weak correlation. Binary logistic regression was used to determine which organ involvement was the most predictive factor for the development of joint and fascial cGVHD. *P* values <0.050 were considered statistically significant. Data were analyzed using StatsDirect Ltd StatsDirect statistical software (*http://www.statsdirect.com*).

## Results

### Patient demographic and clinical history

The study included 32 patients suspected of having developed cGVHD after alloHSCT. 29 patients (28 adult, 1 child) met the criteria for the cGVHD diagnosis according to the NIH cGVHD Consensus Criteria, and all had at least one organ involved. 3 patients were excluded from the analysis as the interdisciplinary panel determined that they had late acute GVHD or an inconclusive diagnosis of cGVHD. This group was smaller than the overall number of patients treated for cGVHD at the University Hospital Centre Zagreb because all patients required functional capacity measures (2- minute walk test and hand grip strength) and all of them had to be examined by a rehabilitation medicine specialist; this is a time-consuming process that was not available to every patient. Patients were not selected for this study based on any criteria other than availability of team members to provide this comprehensive assessment.

The median age at enrolment for group with joint and fascial manifestations was 44.5 years (interquartile range, IQR, 26.25-56.75 years), and 33.3% patients were female (Table 2, Table 3). The median time from transplantation to protocol entry was 843 days (IQR, 343.5- 3986.5 days). The duration of joint and fascia score (JFS) group was 492 days (IQR, 80- 3393.25 days) (Table 3). The most common indications for transplantation in JFS group were acute leukemia or myelodysplastic syndrome (N = 6; 50%), chronic myelogenous leukemia (N = 5; 41.7%), and aplastic anemia in a single patient (N = 1; 8.3%). The majority of JFS patients (N = 7, 58.3%) were subjected to myeloablative conditioning regimen and 5 patients (41.7%) to reduced intensity conditioning regimen. No patients were subjected to high-dose total body irradiation conditioning. The majority of patients were related to their donor (N = 7; 58.3%) and there were 5 (41.7%) male HSCT recipients with female donors. The most common concurrent organ involved in the JFS group was the skin (N = 10; 83.3%), followed by the lungs and eyes, (N = 7; 58,3%), mouth (N = 5; 41.7%), and others (Table 2). The majority of patients in the JFS group (N = 8; 66.7%) had a NIH cGVHD global score deﬁned as “severe.” There were no significant differences in demographic and clinical characteristic between the group with joint and fascia manifestations and without joint and fascia manifestations but with other cGVHD organ system involvement (Table 2).

**Table 2:** Demographic and clinical characteristic of groups with cGVHD*

		Joint/fascia manifestation absent at enrolment N = 17		Joint/fascia manifestation present at enrolment N = 12		*P^†^*
Sex of patient: n (%)	male	7	41.2	8	66.7	0.264
	female	10	58.8	4	33.3	
Main disease: n (%)	ALL, AML and MDS	11	64.7	6	50.0	0.617
	CML (and myeloproliferative disorders)	3	17.6	5	41.7	
	CLL	1	5.9	0	0.0	
	aplastic anemia/PNH	2	11.8	1	8.3	
Conditioning regimen: n (%)	myeloablative	10	58.8	7	58.3	0.999
	reduced intensity	6	35.3	5	41.7	
	unknown	1	5.9	0	0.0	
Donor relationship: n (%)	related	10	58.8	7	58.3	0.999
	unrelated	7	41.2	5	41.7	
Sex mismatch: n (%)	M/M	3	17.6	3	25.0	0.449
	F/M	3	17.6	5	41.7	
	F/F	5	29.4	2	16.7	
	M/F	5	29.4	1	8.3	
	unknown	1	5.9	1	8.3	
Stem cell source: n (%)	peripheral blood	9	52.9	8	66.7	0.827
	bone marrow	7	41.2	4	33.3	
	unknown	1	5.9	0	0.0	
	none	9	56.3	5	41.7	
Intensity of immunosuppression (Sandy's scale)	low	1	6.3	0	0.0	0.289
	moderate	5	31.3	3	25.0	
	high	1	6.3	4	33.3	
Acute GVHD: n (%)	yes	14	82.4	9	75.0	0.242
	no	2	11.8	3	25.0	
	unknown	1	5.9	0	0.0	
cGVHD onset type: n (%)	*de novo*	2	11.8	3	25.0	0.454
	progressive	5	29.4	4	33.3	
	quiescent	10	58.8	4	33.3	
	unknown	0	0.0	1	8.3	
cGVHD organ involvement: n (%)	skin	4	23.5	10	83.3	0.576
	ocular	10	58.8	7	58.3	
	mouth	5	29.4	5	41.7	
	lung	9	52.9	7	58.3	
	liver	5	29.4	4	33.3	
	GI tract	2	11.8	1	8.3	
	genital (women only)	5	29.4	2	16.7	
NIH cGVHD global score: n (%)	mild	1	5.9	0	0.0	0.352
	moderate	9	52.9	4	33.3	
	severe	7	41.2	8	66.7	

*ALL – acute lymphoblastic leukemia; AML – acute myelogenous leukemia; cGVHD – chronic graft- vs -host disease; CLL – chronic lymphocytic leukemia; CML – chronic myelogenous leukemia; F – female; GI – gastrointestinal; M – male; MDS – myelodysplastic syndrome; NIH – National Institutes of Health; PNH – paroxysmal nocturnal hemoglobinuria.

†Fisher exact test or Fisher-Freeman-Halton exact test of independence when the contingency table is larger than 2 × 2.

**Table 3:** Differences in quantitative clinical characteristics of groups with cGVHD*

	Joint/fascia cGVHD absent			Joint/fascia cGVHD present			*P^†^*
	N = 17			N = 12			
	25th percentile	Median	75th percentile	25th percentile	Median	75th percentile	
2- minute walk test	189.50	201.00	211.50	146.75	188.00	202.00	0.097
HGS-D	40.00	46.60	67.45	34.88	48.30	64.98	0.842
HGS-ND	36.95	46.60	53.30	35.50	45.00	60.00	0.859
P-ROM scale	25.00	25.00	25.00	18.50	22.00	22.75	<0.001
NIH total score	3.00	4.00	4.00	4.50	6.50	8.75	0.002
Number of involved organs	2.00	2.00	3.00	3.00	4.00	6.00	0.006
Age at entry	26.00	37.00	52.00	26.25	44.50	56.75	0.565
Days from transplant to enrolment	263.50	574.00	3052.50	343.50	843.00	3986.50	0.364
Days from cGVHD Dx to enrolment	28.25	153.50	452.75	80.00	492.00	3393.25	0.131
Karnofsky/Lansky score	90.00	100.00	100.00	62.50	80.00	87.50	<0.001

*cGVHD – chronic graft-vs-host disease; HGS-D/HGS-ND – hand grip strength (dominant and non-dominant hand); NIH – National Institutes of Health; P-ROM – photographic range of motion scale.

†Mann-Whitney U test.

12 of 29 patients (41.3%) manifested joint and fascial cGVHD according to NIH Consensus Criteria. Among patients who had a NIH joint/fascia score ≥1, 6 had mild disease (50%), 3 (25%) had moderate disease, and 3 (25%) had severe disease. Reduction in ROM among cGVHD patients who were classified as having a NIH joint/fascia score ≥1 was mostly present in wrists/fingers (31%), elbows (31%), ankles (28%), and shoulders (24%). Limitations in ROM were present in multiple joints for 75% of the patients, with limited ROM in at least one joint for patients in JFS group. The involvement of upper extremities was more severe (ROM movement reduction – 66.7%) than the involvement of lower extremities (ROM movement reduction – 25%) for JFS group. Limitations in ROM were most frequently moderate to severe in joints according to the P-ROM score (ie, score 6-5 for shoulders, 6-4 for elbows, wrists/fingers from 6-2, and score 3- 1 for ankles). The median of P-ROM total score at enrollment was 22 (IQR, 18.5- 22.75) for the JFS group, and 25 (IQR, 25- 25) for the group without joint/fascia manifestations (Table 3). All patients with skin involvement in the JFS group had ROM restriction. The involvement of upper extremities was more severe (ROM movement reduction – 60%) than the involvement of lower extremities (ROM movement reduction – 30%) for patients with skin involvement in the JFS group. Patients who had erythematous skin changes or mild dermal sclerotic changes had mild ROM reduction, and were scored as mild NIH joint/fascia score with one or two joints affected. Patients with moderate NIH joint/ fascia score all had dermal sclerotic changes and had more than one joint affected. Patients with deep sclerosis changes (N = 3, 25%) had a severe NIH joint/fascia score and multiple joint involvement.

### Clinical, functional, and laboratory parameters

Our results showed there was a significant positive correlation between cGVHD skin involvement (rho = 0.712; *P* < 0.001), total NIH score (rho = 0.603; *P* = 0.001), number of organs involved with cGVHD (rho = 0.452; *P* = 0.014), C3 complement component (rho = 0.405; *P* = 0.045), white blood count (rho = 0.406; *P* = 0.032) and NIH joint/fascia score. There was a significant negative correlation between P-ROM scale (rho = -0.989; *P* < 0.001), 2-minute walk test (rho = -0.444; *P* = 0.16), serum albumin value (rho = -0.402; *P* = 0.047), Karnofsky score (rho = -0.759; *P* < 0.001), creatine kinase value (rho = -0,462; *P* = 0.012) and NIH joint/fascia score. There was also a significant negative correlation between percentage of cytotoxic T cells CD3+/CD8+ (rho = -0.456; *P* = 0.022) and NIH joint/fascia score, but between cytotoxic T cells CD3+/CD8+ count (rho = -0.378; *P* = 0.062) and NIH joint/fascia score the result approached but did not reach statistical significance (Table 4).

**Table 4:** Correlations between NIH joint/fascia score and significant variables in the group of cGVHD patients: Spearman correlation coefficients (n = 29)*

		NIH joint/fascia score
P-ROM	Correlation coefficient	-0.989
	*P*	<0.001
2- minute walk test	Correlation coefficient	-0.444
	*P*	0.016
NIH score skin	Correlation coefficient	0.712
	*P*	<0.001
NIH total score	Correlation coefficient	0.603
	*P*	0.001
Number of organs involved with cGVHD	Correlation coefficient	0.452
	*P*	0.014
Karnofsky/Lansky score	Correlation coefficient	-0.759
	*P*	<0.001
Cytotoxic T cells (CD3+/CD8+) (%)	Correlation coefficient	-0.456
	*P*	0.022
Cytotoxic T cells (CD3+/CD8+) (number of cells)	Correlation coefficient	-0.378
	*P*	0.062
CK	Correlation coefficient	-0.462
	*P*	0.012
WBC	Correlation coefficient	0.406
	*P*	0.032
C3 comp	Correlation coefficient	0.405
	*P*	0.045
Albumin	Correlation coefficient	-0.402
	*P*	0.047

*C3 – complement component 3; cGVHD – chronic graft-vs-host disease; CK – creatine kinase; NIH – National Institutes of Health; P-ROM– photographic range of motion scale; WBC– white blood count.

Patients with joint and fascial cGVHD more frequently had skin involvement than patients without joint and fascial cGVHD, which was also confirmed by correlation analysis (*P* < 0.001) (Table 5). There was no significant association in other cGVHD organ involvement (eyes, lungs, gastrointestinal tract, liver, mouth, genitalia in female patients) between these two groups (Table 5).

**Table 5:** Differences between investigated groups in NIH cGVHD scoring*

NIH score		Joint/fascia cGVHD absent		Joint/fascia cGVHD present		*P†*
		N	%	N	%	
Genital tract (women only)	0	5	50.0	2	50.0	1.000
	1	1	10.0	1	25.0	
	3	4	40.0	1	25.0	
Lung	0	8	47.1	3	30.0	0.867
	1	5	29.4	5	50.0%	
	2	2	11.8	1	10.0	
	3	2	11.8	1	10.0	
Liver	0	12	70.6	7	63.6	0.894
	1	2	11.8	3	27.3	
	2	2	11.8	1	9.1	
	3	1	5.9	0	0.0	
GI tract	0	15	88.2	11	91.7	1.000
	1	2	11.8	1	8.3	
Eyes	0	7	41.2	5	41.7	0.202
	1	9	52.9	3	25.0	
	2	1	5.9	2	16.7	
	3	0	0.0	2	16.7	
Mouth	0	12	70.6	6	54.5	0.686
	1	1	5.9	2	18.2	
	2	3	17.6	3	27.3	
	3	1	5.9	0	0.0	
Skin	0	13	76.5	2	16.7	<0.001
	1	3	17.6	2	16.7	
	2	1	5.9	3	25.0	
	3	0	0.0	5	41.7	

*cGVHD – chronic graft-vs-host disease; GI – gastrointestinal; NIH – National Institutes of Health.

†Fisher exact test or Fisher-Freeman-Halton exact test of independence when the contingency table is larger than 2 × 2.

Among categorical outcomes, there was no significant association between donor sex, main disease, stem cell source, relationship of donor to patient (related or unrelated), sex mismatch of HSCT, myeloablative conditioning, history of acute cGVHD, greater intensity of immunosuppressive therapy at time of evaluation with joint and fascial cGVHD (Table 2).

Patients with joint and fascial cGVHD had significantly lower Karnofsky score (*P* < 0.001), lower P-ROM scale (*P* < 0.001), higher NIH total score (*P* = 0.002) (Table 3) and lower percentage of cytotoxic T cells CD3+/CD8+ (*P* = 0.017) and cytotoxic T cells CD3+/CD8+ count (*P* = 0.037) than patients without joint and fascial cGVHD (Supplementary Table 1)(web extra material 1).

There was no significant difference between two groups of patients in patients age, longer time from transplant to enrolment in the study, longer time from diagnosis of cGVHD to enrolment in the study, number of organs involved with cGVHD, HGS (dominant and non-dominant hand) (Table 3), laboratory parameters (CRP, platelet count, red blood count, white blood count, lymphocyte count, absolute neutrophil, eosinophil, basophil and lymphocyte subset counts (CD4 T-cell, CD8 T-cell, B-cell, natural killer cell), hemoglobin, C3 and C4 complement component, IgA, IgM, IgG, total proteins, erythrocyte sedimentation rate, ferritin, β2 microglobulin, positive antinuclear antibodies (ANAs), rheumatoid factor, cardiolipin antibodies IgM, cardiolipin antibodies IgG titers and patients who had joint and fascial cGVHD (Supplementary Table 1)(web extra material 1).

### Logistic regression predictive models of cGVHD joint/fascia compared with other organ systems

To determine which organ involvement is the most predictive factor for the development of joint and fascial cGVHD, a binary logistic regression model was used. NIH joint/fascia score was compared with that of other organ systems; with exception of the genital tract, which was excluded because these data were available only for female patients. This model showed that cGVHD skin involvement was a significant predictor (odds ratio, 7.79; 95 confidence interval, 1.87-32.56; *P* = 0.005) of cGVHD joint/fascia involvement, controlled for all other variables included in regression model (Table 6). Regression model was statistically significant (*P* = 0.007) with 65.7% of dependent variable (positive cGVHD joint/fascia involvement) explained. Hosmer-Lemeshow goodness of fit test result for this regression model was not significant (*P* = 0.542), supporting the regression model.

**Table 6:** Predictive models for cGVHD of joint/fascia using NIH scoring system: binary logistic regression*

NIH score	Odds ratio	95% confidence Interval		*P*
		lower	upper	
Skin	7.80	1.87	32.56	0.005
Lung	1.34	0.31	5.72	0.692
Liver	0.24	0.03	1.92	0.179
GI tract	0.05	0.00	3.15	0.158
Eyes	0.89	0.13	5.95	0.902
Mouth	3.78	0.55	26.20	0.178

*cGVHD – chronic graft-vs-host disease GI – gastrointestinal; NIH– National Institutes of Health.

## Discussion

This study showed a 41.3% incidence of joint and fascial manifestations in patients with cGVHD and a strong correlation of joint and fascial cGVHD with skin cGVHD, which was also found in some other studies (9,11,23). Joint/fascia manifestations are significantly associated with skin cGVHD changes, particularly superficial/erythematous and deep skin sclerosis. Patients in this study who were graded as having a severe (N = 3, 25%) NIH joint/fascia score were the only ones with deep skin sclerosis. Upper extremities were more affected with ROM reduction in our study group than lower extremities, consistent with other studies (6,23).Two patients from JFS group had isolated joint involvement (N = 2, 16.7%) without any skin manifestation of cGVHD with both patients graded as having mild NIH joint/fascia score. This percentage is low and consistent with other studies that suggest that isolated joint involvement in cGVHD is fairly uncommon (8).

Since these patients have a high risk for functional impairment, valid and practical methods are needed for assessing functional capacity measures. We found that joint and fascial cGVHD correlated with a worse score on the 2-minute walk test, but not with HGS. This supports findings of some previous studies. One study found an association of 2- minute walk test and cGVHD severity and mortality, although the test was not sensitive to cGVHD response and there was no association of change in 2-minute walk test with subsequent mortality (24). Our data do not support the use of the HGS as a measurement in joint and fascial cGVHD patients (15,24), The 2014 NIH Consensus Development Project for cGVHD also recommended that HGS should not be included in routine patient assessment (25). We also found no association between joint and fascial cGVHD and HGS, although joint contractures and muscle weakness confound the measurement of HGS (26).

This study also identified that some laboratory indicators of inflammation (white blood count, cytotoxic T cells CD3+/CD8+, and albumin) correlated with joint and fascial cGVHD, suggesting increased inflammation in this patient population (27). This was also the case in some other studies, which identified even more inflammatory parameters to be associated with cGVHD (9,11).

This study found a correlation between lower cytotoxic T cell CD3+/CD8+ percentage and joint and fascial cGVHD. The reason for this is unclear, but could be the presence of a lower proportion of cytotoxic T cells in the blood, as cytotoxic T cells have been shown to relocate into organs affected with cGVHD (27). This explanation assumes on-going tissue inflammation in this patient population.

In our patients, joint and fascial cGVHD correlated with elevated serum C3 complement. This is similar to other studies, in which higher C3 levels were associated with most severe joint and fascia involvement and skin (sclerotic type) changes in patients with cGVHD (9,11). Elevated serum C3 complement was also found in glomerular membranes in patients with cGVHD and nephrotic syndrome (28,29), and deposits of C3 were found at the dermal-epidermal junction in skin biopsies of patients with cGVHD (30,31) and with systemic sclerosis (32).

cGVHD characteristics were also compared between patients with and without joint and fascial manifestations at enrolment. Joint and fascia manifestations were significantly associated with more frequent skin involvement but not with other organ involvement (eyes, lungs, liver, GI tract, mouth, genitalia in female patients). As NIH total score was higher, and as Karnofsky score decreased, there was a higher possibility that cGVHD would also manifest as joint/fascia involvement.

There was also a significant association between lower creatine kinase (CK) levels and joint/fascial cGVHD involvement in our study, which may be due to multiple factors, including muscle breakdown from decreased physical activity, increased prednisone use due to disease severity, and as a general indicator for inflammation.

Acute GVHD is an established risk factor for the development of cGVHD (5,15,33), however our study found no correlation between acute GVHD and cGVHD joint involvement. Research that explored skin changes has also not found any significant association between predominantly sclerotic skin changes and acute cGVHD (9,11).

Other potential risk factors considered when examining patients with cGVHD, including use of high-dose total body irradiation conditioning, longer duration from transplantation to enrolment and prevalent cases, were not found to be associated with cGVHD joint/fascia involvement in our patients. This a potential weakness of the study, as other studies have found significant association between this parameters and cGVHD joint/fascia manifestations (6,8).

In the interpretation of our study results, several limitations must be considered. The sample size was relatively small, limiting the statistical analysis with numerous variables, so further investigations with a larger sample size should be considered. Furthermore, patients were only evaluated at one time point, so monitoring of these patients for symptom progression or regression was not possible. Therefore, we cannot conclude that the observed relationships are solely the result of cGVHD. Furthermore, it is unclear if some of the functional impairment are a result of cGVHD treatment– often prednisone, which can cause debilitating myopathy and orthopedic complications (34) or of the disease process itself. However, such in-depth multifaceted studies of cGVHD patients are exceptionally rare.

This study did, however, show a correlation between specific laboratory findings and clinical features with joint and fascial cGVHD. Therefore, we believe that it ﬁlls an important gap in the knowledge about disabling joint and fascial involvement in patients who have developed cGVHD after alloHSCT. AlloHSCT recipients should be carefully monitored in order to identify patients at an earliest stage of the disease and ensure successful therapy interventions. .
