# Supplementary material for: Joint and fascial chronic graft-vs-host disease: correlations with clinical and laboratory parameters
Source: Croat Med J. 2016 Jun;57(3):266–75. doi: 10.3325/cmj.2016.57.266 (PMC4937232; doi:10.3325/cmj.2016.57.266)
Supplement: Supplementary Table 1 [file CroatMedJ_57_s001.pdf]

Supplementary Table 1. Differences in quantitative laboratory measurements of groups with cGVHD

	Joint/fascia cGVHD absent N=17			Joint/fascia cGVHD present N=12			*P***
	25th percentile	Median	75th percentile	25th percentile	Median	75th percentile	
ESR*	11.500	28.000	57.000	17.500	28.000	56.000	0.577
CRP*	1.05	3.00	8.65	2.05	6.95	13.40	0.177
T cells - (%)	69.75	78.00	86.50	42.00	62.00	81.00	0.055
T cells - (number of cells)	1053.25	1290.00	2710.75	468.00	741.00	1760.00	0.112
Helper T cells - (%)	15.00	20.50	38.00	14.00	25.00	38.00	0.742
Helper T cells - (number of cells)	250.75	465.50	651.75	227.00	287.00	672.00	0.642
Cytotoxic T cells (CD3+/CD8+) (%)	38.50	54.50	65.25	17.00	24.00	41.00	0.017
Cytotoxic T cells (CD3+/CD8+) (number of cells)	576.50	942.00	1963.00	227.00	334.00	1261.00	0.037
B cells (%)	6.50	9.50	20.25	8.00	16.00	37.00	0.179
B cells (number of cells)	104.25	214.50	446.25	114.00	347.00	558.00	0.584
NK* cells (%)	5.75	8.00	12.00	8.00	12.00	19.00	0.105
NK* (number of cells)	87.75	174.50	262.50	121.00	174.00	408.00	0.529
RBC*	3.64	4.08	4.60	3.42	4.35	4.89	0.626
Hemoglobin	120.50	130.00	146.00	113.75	136.00	144.50	0.965
Reticulocytes	35.00	55.00	65.75	41.00	50.00	64.00	0.863
Leukocyte	4.75	6.30	8.10	5.90	8.25	10.40	0.099
Monocytes	0.39	0.51	0.84	0.66	0.75	0.83	0.145
Neutrophils	2.06	3.16	4.90	2.02	3.72	5.84	0.626
Lymphocytes	1.53	2.11	3.63	1.08	1.82	2.63	0.268
Eosinophils	0.09	0.15	0.30	0.06	0.24	0.96	0.816
Basophils	0.04	0.06	0.10	0.03	0.05	0.08	0.456
Platelets	148.00	201.00	256.00	149.25	240.50	311.50	0.438
Rheumatism factor	10.00	10.00	13.05	10.00	10.00	12.18	0.286
C3 comp*	0.99	1.14	1.35	1.16	1.34	1.55	0.063
C4 comp*	0.20	0.25	0.29	0.21	0.28	0.38	0.359
ANA*	0.00	0.00	1.00	0.00	1.00	1.00	0.065
Cardiolipin antibodies IgG	5.00	8.00	13.25	7.75	11.00	32.00	0.126
Cardiolipin antibodies IgM	5.00	7.00	9.25	6.00	9.50	25.75	0.277
Beta 2 microglobulin	1.89	2.30	4.05	1.95	2.43	3.37	0.912
Ferritin	157.08	467.50	1281.30	84.50	285.30	706.60	0.348
Total proteins	61.50	70.50	73.50	62.00	65.00	73.50	0.500
IgG*	6.68	8.90	10.90	6.08	8.76	10.97	0.825
IgA*	0.52	1.19	1.90	0.24	0.52	1.80	0.232
IgM*	0.43	1.06	1.24	0.52	0.78	0.99	0.330
Albumin	39.00	42.70	45.00	34.28	37.80	43.03	0.059
CK*	71.50	84.00	128.00	31.50	52.00	72.25	0.018

*ANAs- antinuclear antibodies; C3- complement component 3; C4- complement component 4; cGVHD- chronic graft-versus-host disease; CK – creatine kinase; CRP- C-reactive protein; ESR-

erythrocyte sedimentation rate; IgA- immunoglobulin A; IgG- immunoglobulin G; IgM- immunoglobulin M; NK- natural killer cell; RBC- red blood count; WBC- white blood count.

** Mann-Whitney U test.
